# Incidence and clinical outcomes of diabetes mellitus in HIV-infected adults in Thailand: a retrospective cohort study

**DOI:** 10.1186/s12889-018-5967-7

**Published:** 2018-08-30

**Authors:** Ninutcha Paengsai, Gonzague Jourdain, Romanee Chaiwarith, Apichat Tantraworasin, Chureeratana Bowonwatanuwong, Sorakij Bhakeecheep, Tim Roy Cressey, Jean Yves Mary, Nicolas Salvadori, Natapong Kosachunhanun

**Affiliations:** 1 Clinical Epidemiology Program, Faculty of Medicine, Chiang Mai University, 110 Intavaroros Road, Tambon Sripoom, Muang, Chiang Mai, 50200 Thailand; 2 National Health Security Office (NHSO), Building B 120 Moo 3 Chaengwattana Road, Lak Si District, Bangkok, 10210 Thailand; 3 Institut de recherche pour le développement (IRD) UMI 174-PHPT, 187/10, Changklan Rd, Changklan, Muang, Chiang Mai, 50100 Thailand; 4 Faculty of Associated Medical Sciences, Chiang Mai University, 110 Intavaroros Road, Tambon Sripoom, Muang, Chiang Mai, 50200 Thailand; 5 Harvard T.H. Chan School of Public Health, Boston, MA USA; 6 Division of Endocrinology, Department of Medicine, Faculty of Medicine, Chiang Mai University, 110 Intavaroros Road, Tambon Sripoom, Muang, Chiang Mai, 50200 Thailand; 7 Department of Surgery, Faculty of Medicine, Chiang Mai University, 110 Intavaroros Road, Tambon Sripoom, Muang, Chiang Mai, 50200 Thailand; 8 Department of Medicine, Faculty of Tropical Medicine, Mahidol University, 420/6 Ratchawithi Road, Ratchathewi, Bangkok, 10400 Thailand; 9 National Health Security Office Chiang Mai Branch (Region 1), 6 Mahidol road, Suthep, Muang, Chiang Mai, 50200 Thailand; 10 Department of Molecular & Clinical Pharmacology, University of Liverpool, Liverpool, UK; 11 INSERM UMR 1135, Equipe ECSTRA, Centre de Recherche Epidémiologie Biostatistique Sorbonne Paris Cité, Université Paris Diderot, Paris, France

**Keywords:** HIV infection, Antiretroviral treatment, Diabetes mellitus, Incidence, Diabetic complications

## Abstract

**Background:**

Since 2005, Thailand has scaled up one of the largest antiretroviral treatment (ART) programs in South East Asia. Although diabetes mellitus (DM) incidence is increasing in low and middle-income countries, its burden and contributing factors in the HIV infected population are not well known.

**Methods:**

Using the Thai National AIDS Program data over a period of 8-years, we identified patients diagnosed with DM based on the following records: 1) fasting plasma glucose equal to or greater than 126 mg/dl following the 2013 American Diabetes Association criteria or 2) diagnosis codes E11-E14 of the 2010 WHO International Classification of Diseases, or 3) anti-diabetic drugs. Incidence was the number of new cases divided by that of person-years of follow-up (PYFU). Competing risks survival regression, treating death without DM as a competing event, was used to identify factors associated with DM. The risk of death in patients diagnosed with DM was estimated using Cox regression models.

**Results:**

Data of 763,666 PYFU from 199,707 patients (54.2% male; median age 36.2 years at registration with the program) were available and 8383 cases were diagnosed with DM, resulting in an incidence rate of 11.0 per 1000 PYFU. New DM diagnosis was more likely in men (adjusted sub-distribution hazard ratio 1.2), older patients (compared to patients 18 to 34 years old: 1.8 for 35 to 44; 3.0 for 45 to 59; 3.8 for ≥60), and if ART was initiated (1.3). In 2014, 1313 (16.6%) of 7905 diabetic patients had DM complications (11.5% microvascular complications and 6.9% macrovascular complications). Patients diagnosed with DM were at higher risk of death compared to the others.

**Conclusions:**

DM incidence was higher in this Thailand cohort of HIV infected adults than in the general population. Risk factors were similar to those in the general population, in addition to starting ART.

**Electronic supplementary material:**

The online version of this article (10.1186/s12889-018-5967-7) contains supplementary material, which is available to authorized users.

## Background

The burden of diabetes prevalence is rising especially in low and middle-income countries [1]. In 2017, the International Diabetes Federation estimated that 425 million people worldwide, or 8.8% of adults 20–79 years, were affected by diabetes type 1 and type 2 (8.3% in Thailand) and that half (50.0%) of all people 20–79 years with diabetes are unware of their disease [2].

Known risks factors of diabetes mellitus (DM) include older age, male sex, family history of DM, alcohol use, adiposity, dyslipidemia, hypercholesterolemia, and hypertriglyceridemia [1]. Indeed, hyperlipidemia, insulin resistance and lipodystrophy are commonly observed in HIV-infected patients on antiretroviral treatment (ART) and several studies have suggested that HIV infection and/or antiretroviral drugs may increase the risk of DM [3–6]. The Thai national AIDS program database, which includes data on DM incidence, related mortality and prevalence of related complications, provided a unique opportunity to assess the importance of DM in a South-East Asian HIV infected adult population.

Since Fiscal Year (FY) 2005 (October 1, 2004 to September 30, 2005), Thailand has provided free health services, including ART and laboratory monitoring to HIV-infected patients through the Thailand National AIDS Program (NAP), under the National Health Security Office (NHSO). At each visit, or at the end of each month for hospitalizations, data from HIV-infected patients covered by these schemes are entered into the NAP database. We estimated the incidence of DM diagnosis, investigated associated risk factors and clinical outcomes using the data from adults registered with the NAP under the Universal Coverage Scheme (UCS), which covers about three-quarters of the Thai population [7].

## Methods

This study was a retrospective cohort study and an analysis of secondary data of the NHSO. A total of 199,707 HIV infected adults (≥18 years old) with no history of DM registered between FY2007 to FY2013 and follow up until September 30,2014 (end of FY2014) for care with the NAP and received care under UCS in 1035 hospitals throughout Thailand.

We extracted the following patient characteristics from the NHSO database: sex, date of birth, weight, height, fasting plasma glucose (FPG), triglycerides, hepatitis C virus infection, absolute CD4 cell count (including nadir) and date of ART initiation [7]. A patient not showing up for at least 7 months after last visit was considered lost to follow up (LTFU). The number of PYFU was calculated from date of NAP registration (baseline) to censoring date, i.e. 7 months after last visit date, date of death (within 7 months after last visit), date of first DM diagnosis, or September 30, 2014, which ever occurred first.

For this study, DM was considered diagnosed at the first date of at least two of the following records: 1) FPG ≥126 mg/dl following the 2013 American Diabetes Association criteria [8] or 2) confirmed diagnosis codes E11-E14 (which excludes type-1 diabetes mellitus) of the 2010 WHO International Classification of Diseases (ICD-10) [9], or 3) confirmed receipt of anti-diabetic drugs [8]. Hypertriglyceridemia was defined by triglycerides ≥200 mg/dl [4]. DM incidence rates were estimated by the number of new diagnoses divided by the total number of PYFU. The 95% confidence intervals (CIs) were calculated using the quadratic approximation to the Poisson log likelihood [10].

We fitted competing risks survival regression models (Fine-Gray) [11–13], treating death without DM as a competing event (except in case of death after DM diagnosis), to assess the association of new DM diagnosis occurrence with sex, baseline age (categories: 18–34, 35–44, 45–59 or ≥ 60 years [14]) and Body Mass Index (BMI) (≥25 or < 25 kg/m^2^); and baseline and time-updated triglycerides (< 200 or ≥ 200 mg/dl), baseline absolute CD4 cell count (< 200 or ≥ 200 cells/mm^3^), and time-updated ART initiation (yes/no) [4, 14]. All models were adjusted for the existence of at least a previous record of FPG (dichotomous, time-updated variable) and time-updated absolute CD4 < 200 cells/mm^3^. We imputed missing data by multiple imputation with chained equations (MICE procedure, Stata Corp, College Station, Texas, USA) based on logistic regression for binary variables if they were ≤ 20% missing data (time-updated absolute CD4 values if there was a missing data more than 9 months after the previous known value) [15]. We conducted multivariable analyses using a backward selection approach starting with factors significantly associated with time to DM in the univariable analysis (*p* ≤ 0.20), excluding variables with more than 20% missing values (baseline BMI, baseline and time-updated triglycerides, baseline absolute CD4 data) and sensitivity analyses [16] were conducted including each of these variables. The cumulative incidence function (CIF) at time t was defined as the probability of a new diagnosis from baseline using Gray’s sub-distribution hazard technique [17, 18].

A Poisson distribution was assumed to calculate the 95% confidence intervals (CIs) of prevalence. We used the 2010 WHO ICD-10 [9] codes for microvascular diabetic complications: ophthalmic complications code E11-E14 with additional code “.3”, diabetic retinopathy codes H36.0*, renal complications codes E11-E14 with additional external cause code “.2” or code N18, neurological complications codes E11-E14 with additional code “.4” (diabetic amyotrophy, autonomic neuropathy, mononeuropathy, polyneuropathy and autonomic), and macrovascular diabetic complications: ischaemic heart diseases (ICH) codes I00-I25, cerebrovascular disease (CVD) codes I60-I69, peripheral circulatory complication codes E11-E14 with additional code“.5” (diabetic gangrene, peripheral angiopathy or diabetic ulcer), and amputation (The 2010 WHO ICD-9-CM procedure code 84.11, 84.12, 84.14, 84.15 and 84.17 [19]).

We analyzed the interaction between time-updated ART initiation and time-updated DM for the risk of death by year using a constant proportional hazard in univariable and multivariable Cox’s survival regression analyses (*p* ≤ 0.05). We tested the proportional hazards assumption of each model based on Schoenfeld residuals [20] and considered time-updated DM diagnosis as a time-varying variable [21, 22].

Analyses were performed using Stata software, version 13.1 (Stata Corp, College Station, Texas, USA). Patient identifiers were encrypted by NHSO prior data management and analysis. The analysis plan was approved by the Ethical Committee of the Faculty of Medicine, Chiang Mai University, Thailand on March 18, 2014 (114/2014, Research ID: COM-2557-02140).

## Results

### Study population and follow up

All 199,707 HIV-infected adults (54.2% male) with no history of DM, registered with the NAP through the UCS from FY2007 to FY2013, were included in the analysis. Baseline median age was 36.2 years (IQR 30.5 to 42.6), BMI 20.2 kg/m^2^ (IQR 18.2 to 22.4), absolute CD4 cell count 120 cells/mm3 (IQR 33 to 324) and nadir CD4 cell count 119 cells/mm^3^ (IQR 33 to 323); and 1408 of 199,707 (0.7%) had a history of hepatitis C infection. Of the 11,763 patients with available records, 2511 (21.4%) had triglycerides ≥200 mg/dl.

**Fig. 1:**
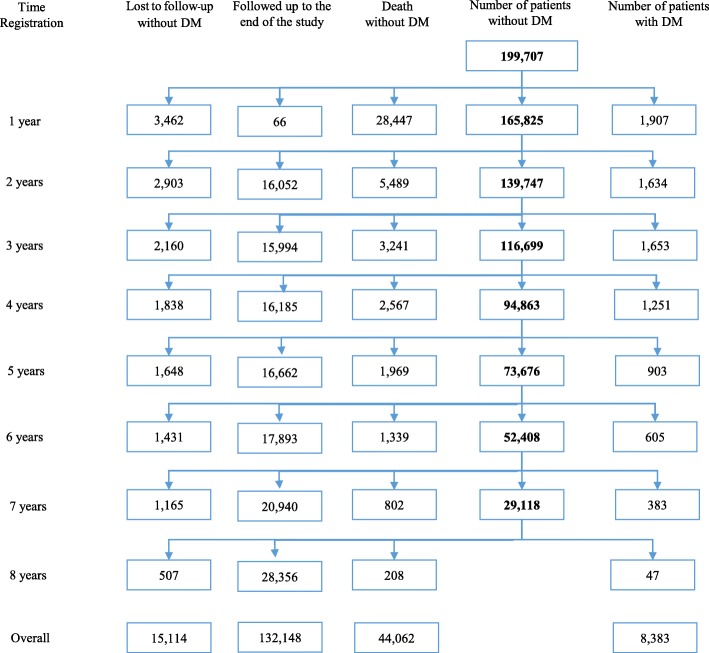
Study population for the analysis of incidence of type-2 diabetes mellitus (DM) in the National AIDS Program in Thailand

Data from a total of 763,666 PYFU were available from FY2007 to FY2014 (median follow-up time was 3.8 years [IQR 1.6 to 6.1]); 15,114 patients (7.6%) were lost-to-follow up, 8383 (4.2%) were diagnosed with DM and 44,062 (22.1%) died (Fig. 1). 116,394 patients (58.3%) had at least one FPG measure. ART was initiated in 152,664 patients (76.4%) and the median duration from registration to ART initiation was 1.3 months (IQR 0.7 to 4.9). Of note, 24,539 (55.7%) of the patients who died had not initiated ART.

### Patients diagnosed with DM

**Fig. 2:**
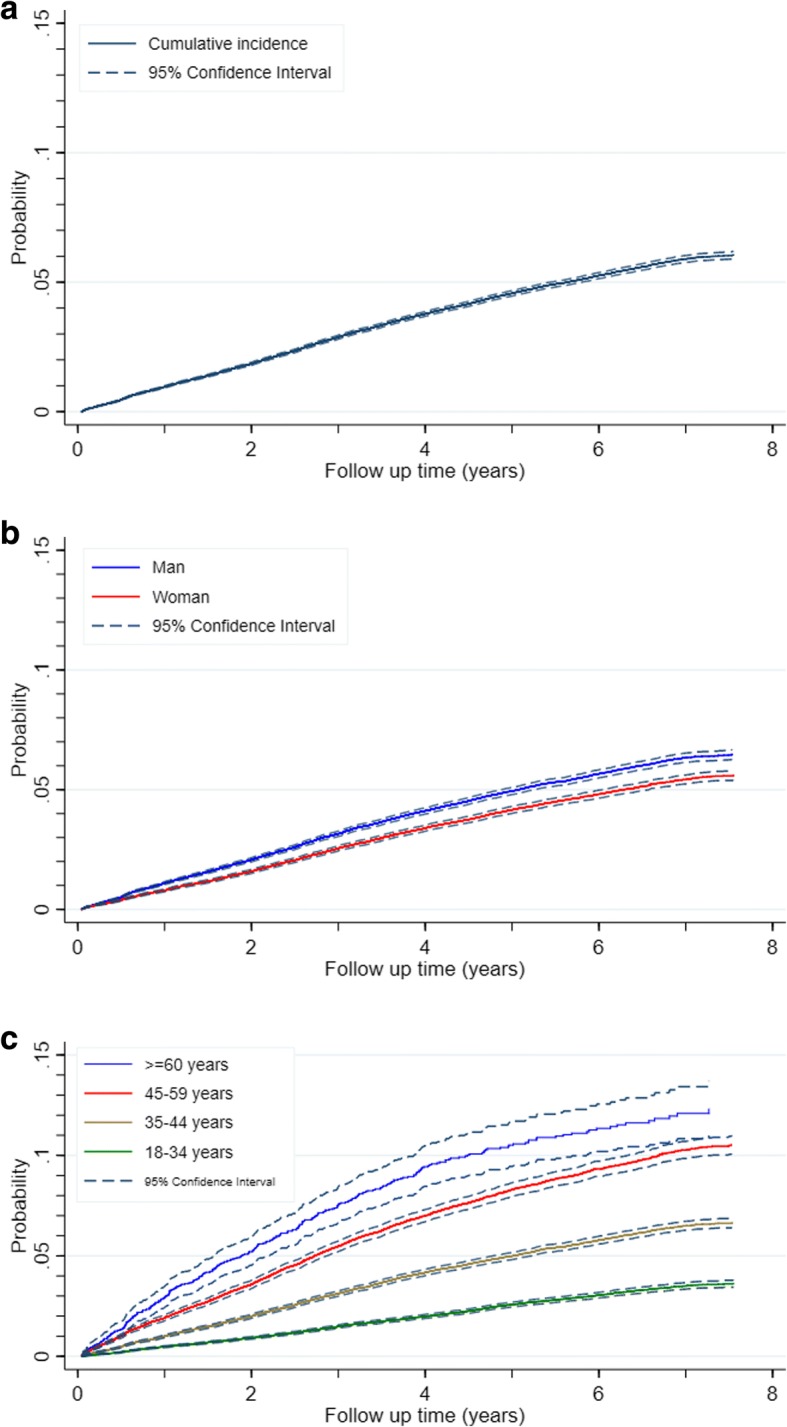
The estimate cumulative incidence function of diabetes mellitus in HIV-infected adults (**a**) overall (**b**) by sex and (**c**) by age at baseline. Legend text: (**a**) Cumulative incidence, 95% Confidence Interval (**b**) Man, Woman (**c**) > = 60 year, 45–59 year, 35–44 years, 18–34 year, 95% Confidence Interval

Over the study period, 8383 patients were diagnosed with DM leading to an estimated cumulative incidence of DM of 11.0 per 1000 PYFU (CI 10.7 to 11.2). The incidence rate of DM was 10.8 per 1000 PYFU during the first year, reached 13.0 per 1000 PYFU during the third year and tended to decrease thereafter. The incidence was 12.5 per 1000 PYFU in men (CI 12.2 to 12.9) and 9.5 per 1000 PYFU in women (CI 9.1 to 9.8) (*p* < 0.001). Male sex, age above 35 years, baseline BMI ≥25 kg/m^2^, triglycerides ≥200 mg/dl, absolute CD4 cell count < 200 cells/mm^3^ and hepatitis C infection were associated with a higher incidence rate of DM (Table 1). The estimated cumulative incidence of DM was 3.8% (CI 3.7 to 3.9) at 4 years follow-up. It was about 3.4% (CI 3.3 to 3.5) in women and 4.1% (CI 4.0 to 4.3) in men; 2.0% (CI 1.9 to 2.1) in patients aged 18–34 years, 4.2% (CI 4.0 to 4.3) in patients aged 35–44 years, 7.0% (CI 6.7 to 7.3) in patients aged 45–59 years and 9.4% (CI 8.5 to 10.5) in patients aged ≥60 years (Fig. 2).

**Table 1:** Incidence of diabetes mellitus according to patient baseline characteristics

Baseline characteristics	Category	Total	Number of patients with diabetes mellitus	PYFU	Incidence per 1000 PYFU	95% CI	*P* value*
Total		199,707	8383	763,666	10.98	10.74–11.21	
Fiscal year of registration	2007	41,537	2852	248,385	11.48	11.07–11.91	< 0.001
	2008	34,100	1729	165,818	10.43	9.95–10.93	
	2009	28,872	1343	119,249	11.26	10.68–11.88	
	2010	26,026	916	89,424	10.24	9.60–10.93	
	2011	24,270	699	66,880	10.45	9.70–11.26	
	2012	22,861	443	46,229	9.58	8.73–10.52	
	2013	22,041	401	27,682	14.49	13.14–15.98	
Sex	Female	91,407	3604	381,552	9.45	9.14–9·76	< 0.001
	Male	108,300	4779	382,114	12.51	12.16–12.87	
Age (years)	18–34	87,948	2076	354,196	5.86	5.61–6.12	< 0.001
	35–44	74,610	3521	288,739	12.19	11.80–12.60	
	45–59	33,154	2428	110,286	22.02	21.16–22.91	
	≥60	3995	358	10,444	34.28	30.90–38.02	
History of hepatitis C infection	No	198,299	8327	760,649	10.95	10.71–11·18	< 0.001
	Yes	1408	56	3017	18.56	14.28–24.12	
Variables with more than 20% missing data							
Body mass index (kg/m^2^)	< 25	59,249	2154	218,284	9.87	9.46–10.29	< 0.001
	≥25	7296	509	29,241	17.41	15.96–18.99	
Triglycerides (mg/dl)	< 200	9252	289	30,352	9.52	8.48–10.69	0.0021
	≥200	2511	111	8280	13.41	11.13–16.15	
Absolute CD4 cell count (cells/mm^3^)	≥200	36,877	1283	144,795	8.86	8.39–9.36	< 0.001
	< 200	58,570	2114	183,322	11.53	11.05–12.03	
Nadir CD4 cell count (cells/mm^3^)	≥200	36,877	1279	144,522	8.85	8.38–9.35	< 0.001
	< 200	58,671	2118	183,721	11.53	11.05–12.03	

Abbreviations: *CI* confidence interval, *PYFU* Person-years of follow-up

*Chi-square test

Table 2 provides the results of the univariable and multivariable analyses using competing risks survival models. In the univariable analyses, DM diagnosis was associated with male sex, older age at baseline, higher BMI at baseline, hypertriglyceridemia at baseline and time-updated, and time-updated ART initiation (all *p* ≤ 0.05). In the multivariable analysis, DM diagnosis was associated with male sex, age above 35 years at baseline, and time-updated ART initiation. The sensitivity analyses restricted to cases with available baseline BMI and hypertriglyceridemia data showed similar associations with the factors identified in the main analyses (Additional file 1: Table S1).

**Table 2:** Univariable and multivariable competing risk regression analyses of potential risk factors for diabetes mellitus (treating death without diabetes mellitus as a competing event)

Variables	Category	Univariable			Multivariable		
		SHR	[95% CI]	*P* value	aSHR	[95% CI]	*P* value
Sex	Female	1	Reference		1	Reference	
	Male	1.22	1.17–1.27	< 0.001	1.13	1.08–1.18	< 0.001
Age (years) at baseline	18–34	1	Reference		1	Reference	
	35–44	1.88	1.78–1.99	< 0.001	1.83	1.73–1.93	< 0.001
	45–59	3.17	2.99–3.37	< 0.001	3.1	2.92–3.29	< 0.001
	≥60	4.41	3.93–4.94	< 0.001	4.32	3.85–4.84	< 0.001
History of hepatitis C infection at baseline	No	1	Reference		1	Reference	
	Yes	1.24	0.95–1.62	0.106	1.09	0.84–1.43	0.515
Time-updated antiretroviral treatment initiation	No	1	Reference		1	Reference	
	Yes	1.26	1.19–1.35	< 0.001	1.26	1.18–1.34	< 0.001
Baseline body mass index (kg/m^2^) (*n* = 66,529)	< 25	1	Reference				
	≥25	1.97	1.78–2.17	< 0.001			
Baseline triglycerides (mg/dl) (*n* = 11,762)	< 200	1	Reference				
	≥200	1.41	1.14–1.76	0.002			
Baseline absolute CD4 cell count (cells/mm^3^) (*n* = 95,432)	≥200	1	Reference				
	< 200	1.04	0.96–1.12	0.33			
Nadir CD4 cell count (cells/mm^3^) (*n* = 95,476)	≥200	1	Reference				
	< 200	0.89	0.83–0.96	0.002			

Abbreviations: *CI* confidence interval, *n* number of patients with available data, *SHR* sub-distribution hazard ratio, *aSHR* adjusted sub-distribution hazard ratio

Note: impute time-updated CD4 (18.0% of values); Univariable and multivariable analysis were adjusting for previous fasting plasma glucose measurement and time-updated absolute CD4 < 200 cells/mm^3^

### Prevalence of diabetes mellitus and diabetes-related complications

Of 8383 DM patients, the median follow-up time since DM diagnosis until end of study was 2.7 years (IQR 1.2 to 4.3). In the last year of the study (FY2014), of 157,980 alive patients on follow-up, 7905 patients (5.0%, CI 4.9 to 5.1) had DM. It was higher in men and increased with age. Of 7905, 1313 (16.6%, CI 15.7 to 17.5) patients had DM-related complications. The overall prevalence of microvascular complications was 11.5% (CI 10.8 to 12.3); renal (nephropathy) 7.8% (CI 7.2 to 8.4), ophthalmic 3.1% (CI 2.7 to 3.5) (diabetic retinopathy 0.6% [CI 0.5 to 0.8]), and neurological (neuropathy) 2.1% (CI 1.8 to 2.4). The overall prevalence of macrovascular complications was 6.9% (CI 6.3 to 7.5); ischemic heart diseases 3.1% (CI 2.7 to 3.5), cerebrovascular disease 2.7% (CI 2.3 to 3.1) and peripheral circulatory 1.6% (CI 1.3 to 1.9) (amputation 0.3% [0.2 to 0.5]) (see Table 3).

**Table 3:** Prevalence of diabetes related complications among HIV-infected adults with diabetes during last year of follow-up (Fiscal Year 2014)

Type of complications	Category	Sub category	Number of patients with complications	Total	*P*-value^a^	Annual prevalence (%)	95% CI
MICROVASCULAR OR MACROVASCULAR			1313	7905		16.61	15.72–17.53
	Sex				0.144		
		Female	596	3444		17.31	15.94–18.75
		Male	717	4461		16.07	14.92–17.29
	Age in years				< 0.001		
		18–34	81	770		10.52	8.35–13.07
		35–44	321	2901		11.07	9.89–12.34
		45–59	658	3543		18.57	17.18–20.05
		≥60	253	691		36.61	32.24–41.41
MICROVASCULAR			911	7905		11.52	10.79–12.30
Ophthalmic			246	7905		3.11	2.74–3.53
	Sex				0.122		
		Female	119	3444		3.46	2.86–4.13
		Male	127	4461		2.84	2.37–3.39
	Age in years				0.004		
		18–34	25	770		3.25	2.10–4.79
		35–44	71	2901		2.45	1.91–3.09
		45–59	115	3543		3.25	2.68–3.90
		≥60	35	691		5.07	3.53–7.04
*Diabetic retinopathy*			49	7905		0.62	0.46–0.82
	Sex				0.443		
		Female	24	3444		0.7	0.45–1.04
		Male	25	4461		0.56	0.36–0.83
	Age in years				0.072		
		18–34	2	770		0.26	0.03–0.94
		35–44	13	2901		0.45	0.24–0.77
		45–59	26	3543		0.73	0.48–1.08
		≥60	8	691		1.16	0.50–2.28
Renal			615	7905		7.78	7.18–8.42
	Sex				0.174		
		Female	284	3444		8.25	7.31–9.26
		Male	331	4461		7.42	6.64–8.26
	Age in years				< 0.001		
		18–34	24	770		3.12	2.00–4.64
		35–44	149	2901		5.14	4.34–6.03
		45–59	305	3543		8.61	7.67–9.63
		≥60	137	691		19.83	16.65–23.44
Neurological			163	7905		2.06	1.76–2.40
	Sex				< 0.001		
		Female	101	3444		2.93	2.39–356
		Male	62	4461		1.39	1.07–1.78
	Age in years				< 0.001		
		18–34	4	770		0.52	0.14–1.33
		35–44	32	2901		1.1	0.75–1.56
		45–59	86	3543		2.43	1.94–3.00
		≥60	41	691		5.93	4.26–8.05
MACROVASCULAR			545	7905		6.89	6.33–7.50
Ischaemic heart diseases			243	7905		3.07	2.70–3.49
	Sex				0.681		
		Female	109	3444		3.16	2.60–3.82
		Male	134	4461		3	2.52–3.56
	Age in years				< 0.001		
		18–34	14	770		1.82	0.99–3.05
		35–44	45	2901		1.55	1.13–2.08
		45–59	116	3543		3.27	2.71–3.93
		≥60	68	691		9.84	7.64–12.48
Cerebrovascular disease			212	7905		2.68	2.33–3.07
	Sex				0.002		
		Female	70	3444		2.03	1.58–2.57
		Male	142	4461		3.18	2.68–3.75
	Age in years				< 0.001		
		18–34	10	770		1.3	0.62–2.39
		35–44	45	2901		1.55	1.13–2.08
		45–59	109	3543		3.08	2.53–3.71
		≥60	48	691		6.95	5.12–9.21
Peripheral circulatory			123	7905		1.56	1.29–1.86
	Sex				0.174		
		Female	61	3444		1.77	1.35–2.28
		Male	62	4461		1.39	1.07–1.78
	Age in years				< 0.001		
		18–34	10	770		1.3	0.62–2.39
		35–44	24	2901		0.83	0.53–1.23
		45–59	70	3543		1.98	1.54–2.50
		≥60	19	691		2.75	1.66–4.29
*Amputation*			25	7905		0.32	0.20–0.47
	Sex				0.719		
		Female	10	3444		0.29	0.14–0.53
		Male	15	4461		0.34	0.19–0.55
	Age in years				0.214		
		18–34	4	770		0.52	0.14–1.33
		35–44	5	2901		0.17	0.06–0.40
		45–59	12	3543		0.34	0.18–0.59
		≥60	4	691		0.58	0.16–1.48

^a^Fisher’s exact test

### Diabetes mellitus and mortality

During the study period, from a total of 199,707 patients, 46,252 died, including those who died after a diagnosis of DM. During the first year of follow-up, patients not diagnosed with DM had a higher risk of death than those with DM diagnosis, adjusting for sex, age, time-updated absolute CD4 cell count and time-updated ART initiation, with a strong interaction between time-updated DM and ART initiation (aHR 9.3, CI 6.3 to 13.7 in patients not receiving ART; aHR 2.6, CI 1.7 to 4.0 in patients receiving ART). In the following 3 years, the risk of death was similar in those with and without DM diagnosis (aHR 1.0, CI 1.0 to 1.1) but tended to increase thereafter in patients with DM (5th to 8th year: aHR 1.2, CI 1.0 to 1.5, *p* = 0.05).

## Discussion

The incidence of new DM diagnosis during the follow up of 199,707 of HIV infected adults over 8 years in Thailand (763,666 PYFU) was 11.0 per 1000 PYFU with slight variations over time. The study population included 45.8% females and was relatively young (median 36.2 years), with a lean baseline body shape (median 20.2 kg/m^2^) and a low absolute CD4 cell count (median 120 cells/mm^3^). In multivariable analyses, risk factors associated with DM diagnosis were male sex and older age, which are known risk factors in the general population. In addition, initiation of antiretroviral treatment was associated with DM, as previous reported [14, 23–25].

Our overall DM incidence estimate was similar to or slightly lower than that reported in several studies conducted in population with similar characteristics (sex, age, CD4, ART): 11.35 per 1000 PYFU in a study of 6816 HIV-infected patients registered with the South Carolina Medicaid system [3] in the USA (43% women, median age 39.0 years, and 80.4% on ART); 14.1 per 1000 PYFU (95% CI, 11.6 to 17.0) in the French Aproco-Pilote cohort [6] (78.5% men, median age 37.0 years, median CD4 cell count 280 cells/mm^3^, all on ART, men 14.6 per 1000 PYFU versus women 12.6 per 1000 PYFU); 13.1 per 1000 PYFU in the National Taiwan University Hospital study [26] (86.0% men, median age 34.0 years, median CD4 cell count 92 cells/mm^3^, all patients on ART). The incidence rate of DM and the risk factors for DM in our study were similar to 13.7 per 1000 PYFU, the pooled incidence rate estimated in a meta-analysis [27].

Male sex and older age, which are known factors of DM [1] may account for the differences in estimated incidence reported by previous studies of HIV infected patients, for instance 5.0 per 1000 PYFU in a younger population composed of 76% women in Thailand [28], or 26.0 per 1000 PYFU in older HIV-infected men (median 46 years) participating in the US Multicenter AIDS Cohort Study [29]. ART initiation was identified as a risk factor of DM in our study, similarly to that reported in the Data Collection on Adverse events of Anti-HIV Drugs study (relative risk of 1.1 per year of exposure) [5]. It is possible that, even after adjustment for age and treating death as a competing event, improved survival on ART is a confounder for this association, i.e. patients who survive longer are more likely to develop DM. Also, the risk may vary according to specific antiretroviral drugs [28].

The incidence of DM in HIV-infected adults aged 35 to 59 years in our study was 14.9 per 1000 PYFU, higher than in the Thai general adult population (7.8 to 11.4 per 1000 PYFU) [23, 24], suggesting a contribution of HIV infection or ART in the risk of DM.

At the end of study period, the overall estimated prevalence of DM was lower than in the Thai general population (5.0% versus 8.9%) [30]. This may have resulted from several differences between our population and the general population: lower median age, lower BMI, or regular medical follow-up and FPG assessments in our cohort. Also, DM-related complications seemed less prevalent [7, 30, 31], likely for similar reasons. Generally, microvascular complications develop 5 years after diagnosis [32, 33]. However, with a median follow-up of 2.7 years in our study the prevalence of microvascular complications was 11.5% and the most common was diabetes nephropathy. HIV infection or/and antiretroviral drugs may also have affected the kidneys or accelerated microvascular complications.

Our study showed that the risk of death in patients diagnosed DM tended to be higher after 4 years of follow-up, a finding similar to that reported by a previous study in Thailand [34]. Although causes of death were not available, we hypothesized that the increasing risk of death with DM may have been related to cardiovascular and renal complications as well as HIV infection itself.

A limitation of our study is that the NAP database has been primarily designed for overall monitoring of the program with missing data not systematically tracked as in clinical studies. Importantly, the NAP database represents a unique source of information that likely reflects the actual DM burden in the HIV infected population. Also, the lack of systematic FPG assessments in some patients may have led to an underestimation of DM incidence. This issue is now addressed by a specific program [35] which promotes systematic DM screening in at risk patients [14].

## Conclusion

HIV infected adults in Thailand had a slightly higher incidence of DM than that estimated in general population with similar common risk factors. Antiretroviral treatment may contribute to the risk of DM but to a lesser extent than the common risk factors of DM.

## Additional file

Additional file 1: **Table S1.** Sensitivity analysis for variable with missing data using multivariable competing risk regression analyses of potential risk factors for diabetes mellitus only complete cases (treating death without diabetes mellitus as a competing event). (XLSX 12 kb)

## Abbreviations

aHR: Adjusted hazard ratio
ART: Antiretroviral treatment
aSHR: Adjusted sub-distribution hazard ratio
BMI: Body mass index
CI: Confidence interval
CIF: Cumulative incidence function
DM: Diabetes mellitus
FPG: Fasting plasma glucose
FY: Fiscal year
ICD: International classification of diseases
LTFU: Lost to follow up
MICE: Multiple imputation with chained equations
NAP: The Thailand National AIDS Program
NHSO: The National Health Security Office
PYFU: Person-years of follow-up
SHR: Sub-distribution hazard ratio
UCS: Universal Coverage Scheme
WHO: World Health Organization
